# Hysteria and Certain Allied Conditions

**Published:** 1898-04

**Authors:** 


Hysteria and Certain Allied Conditions: Their Nature and Treatment, with Special Reference to the Application of the Rest Cure, Massage, Electro-Therapy, Hypnotism, etc. By George J. Preston, M. D., Professor of the Diseases of the Nervous System, College of Physicians and Surgeons, Baltimore; Visiting Physician to the City Hospital; Consulting Neurologist to Bay View Asylum, the Hebrew Hospital, the Church Home and Infirmary, etc.; Member of the Medical and Chirurgical Faculty of Maryland, the American Neurological Association, etc. Illustrated. Published by P. Blakiston, Son & Co., 1012 Walnut St., Philadelphia. 1897. Cloth, $2.

This very valuable little work of 298 pages, will prove a profitable investment for any physician. No treatise on the subject has been published for several years, and during this time much has been added to the knowledge of its nature and therapeutics. While the literature of hysteria is vast, the author has gleaned with rare judgment and discretion, and as a result his treatise contains no non-essentials. It is adorned with numerous illustrations, and the text is a model of conciseness and elegance. Beginning with a short historical account of the progress of our knowledge of hysteria from Hippocrates down to the present, he next takes up and thoroughly discusses the nature, etiology and pathology. An entire chapter is devoted to the symptomatology. The whole range of the subject is discussed in eleven chapters, the last three being devoted to the therapeutics. Electro-therapy, massage, the rest cure and hypnotism, are prominently discussed, as therapeutic measures of great value. On the whole, the work is recommended without qualification. J.

